# Isolation, identification and potential probiotic characterization of lactic acid bacteria from Thai traditional fermented food

**DOI:** 10.3934/microbiol.2021026

**Published:** 2021-11-05

**Authors:** Sunisa Suwannaphan

**Affiliations:** Department of Food Science and Technology, Faculty of Agricultural Technology and Agro-Industry, Rajamangala University of Technology Suvarnabhumi, Phra Nakhon Si Ayutthaya, 13000, Thailand

**Keywords:** Thai traditional fermented food, Phra Nakhon Si Ayutthaya, lactic acid bacteria, probiotic bacteria, probiotic properties

## Abstract

The probiotic potential of lactic acid bacteria (LAB) isolated from Thai traditional fermented food was investigated. Forty-two samples were collected from four markets in Phra Nakhon Si Ayutthaya Province. Out of 50 isolated LAB, 6 (a3, f4, f8, K1, K4 and K9) obtained from pla-ra and bamboo shoot pickle samples showed high tolerance to gastrointestinal tract conditions. These isolates were selected to identify and characterize their probiotic properties. Isolate a3 was identified as *Weissella thailandensis*, isolates f4 and f8 were identified as belonging to *Enterococcus thailandicus* and isolates K1, K4 and K9 were determined as *Limosilactobacillus fermentum*. All six LAB exhibited high autoaggregation ability (93.40–95.01%), while *W. thailandensis* isolate a3 showed potential for coaggregation in almost all the pathogenic bacteria tested. Cell-free supernatant (CFS) obtained from all isolates did not inhibit *Staphylococcus aureus*. CFS derived from *L. fermentum* isolate K4 showed the most efficient antimicrobial activity, in particular against Gram-negative bacteria, while *L. fermentum* isolate K4 presented high surface hydrophobicity in the presence of xylene and n-hexane. All LAB isolates were found to be resistant to clindamycin and nalidixic acid, whereas *E. thailandicus* isolate f8 exhibited resistance to most of the antibiotics tested. *L. fermentum* isolate K4 showed promise as a suitable probiotic candidate for future applications in the food industry due to tolerance to gastrointestinal tract conditions with high surface hydrophobicity and inhibited most of the pathogens tested.

## 1. Introduction

Traditionally, fermented foods have been used to preserve and improve food flavor in many Asian countries. Thai traditional fermented foods are made from meat, fish, cereals, legumes, vegetables and fruits by several processes of natural fermentation. They have many health benefits as rich sources of bioactive compounds and probiotics. Two major sources of probiotic microorganisms are the gastrointestinal tract (humans, pigs, rats, poultry, marine and freshwater fishes) and fermented dairy products (kurat, kefir, koumiss, yogurt, sheep and cow milks and cheese). Plant and animal-based fermented foods also offer important alternatives to isolate probiotic bacteria for viable and useful applications [1]. Possible sources of probiotic candidates isolated in Thailand are derived from fermented fruits (buay-dorng, loog-pling-dorng, ma-kaam-dorng and poot-sa-dorng), fermented vegetables (phak-gard-dorng, horm-dorng, hooa-phak-gard-dorng, kra-tiam-dorng, ginger and bamboo shoot pickles), fermented soybeans (see-iu, tao-hoo-yee and tao-jieo), fermented fishes (pla-ra, nam-pla, bu-du, pla-chom, kung-chom, pla-uan and som fak), fermented shrimp and other sea foods (ka-pi, koei-cha-loo, koei-nam, koong som, hoi-kraeng-dorng, hoi-som and poo-khem) and fermented animals (mam, naem and sai-krok-prieo) [2]–[4].

Probiotics are living organisms, and when administrated in sufficient amounts they confer health benefits on the host [5]. Recently, increased interest has been shown in probiotics in view of their recorded safe use and recognized effects on human health. Probiotics have diverse health benefits including promoting good digestion, enhancing resistance to infection, improving the immune system [6], restoring and balancing the gut microflora [7], pathogen interference, as anti-carcinogenic and anti-mutagenic agents and ameliorating the effects of diseases such as diabetes and antibiotic-associated acute diarrhea and constipation [8]. Most probiotic microorganisms emanate from *Lactobacillus* (*L. bulgaricus*, *L. acidophilus*, *L. casei*, *L. paracasei*, *L. helveticus*, *L. lactis*, *L. salivarius*, *L. plantarum*, *L. gasseri*, *L. brevis*, *L. pentosus*, *L. rhamnosus*, *L. coryniformis* and *L. johnsonii*), *Limosilactobacillus* (*L*. *fermentum* and *L. reuteri*), *Bifidobacterium* (*B. animalis*, *B. longum*, *B. breve*, *B. bifidum* and *B. infantis*), *Leuconostoc* (*L. kimchi*, *L. mesenteroides* and *L. fallax*), *Weissella* (*W*. *koreenis*, *W. confusa* and *W. cibaria*), *Streptococcus* (*S. thermophilus*), *Pediococcus* (*P. pentosaceus*), *Enterococcus* (*E. faecalis* and *E. faecium*) and *Bacillus* (*B*. *siamensis*, *B. breve* and *B. clausii*) [1],[9]–[11] as Gram-positive, with the main fermentation product as lactic acid [12]. Several researchers have isolated probiotic bacteria from Thai fermented food such as *L. salivarius* and *L. paracasei* from sai-krog-prieo, *L. brevis* from naem, phak-gard-dorng, som-fak and pla-som, *L. fermentum* from fermented fish, fermented pork and fermented tea leaves, *L. plantarum* from bamboo shoot pickle, phak-gard-dorng and sai-krog-prieo, *P. halophilus* from pla-ra, bu-du, hoi-som and ka-pi and *B. siamensis* from phak-dorng [4],[11]. Probiotic strains possess properties such as tolerance to gastrointestinal conditions (gastric, intestinal and bile acids). Other functional properties used to characterize probiotics are surface hydrophobicity, autoaggregation, coaggregation with pathogenic bacteria, antimicrobial activity and antibiotic susceptibility [13]. Nowadays, screening probiotic bacteria from fermented food has gained increasing attention to obtain new probiotic strains with satisfactory technological properties in the food industry [14]. Therefore, here, probiotic bacteria isolated from Thai traditional fermented food in Phra Nakhon Si Ayutthaya Province were identified and their properties such as tolerance to simulated gastric juice and bile salt, autoaggregation, coaggregation, surface hydrophobicity, antimicrobial activity and antibiotic susceptibility were investigated.

## 2. Materials and methods

### 2.1. Sample collection

Forty-two samples of Thai traditional fermented food such as bamboo shoots and ginger pickles, phak-gard-dorng, pla-ra and Thai fermented pork sausage were collected from four markets in Phra Nakhon Si Ayutthaya Province as Grand, Chao Phrom, DD and 4 Right Development. All samples were stored at 30 °C until required for use.

### 2.2. Isolation of lactic acid bacteria (LAB)

LAB were isolated on MRS agar (HiMedia, India) supplemented with bromocresol purple as an indicator and incubated at 37 °C for 24–48 h. Single bacterial colonies that produced a yellow zone were selected according to morphological differences. Gram-staining and catalase tests were carried out for 135 colonies. Only Gram-positive and catalase negative colonies were sub-cultured in MRS broth and kept in glycerol before experimental use.

### 2.3. Screening for probiotic properties

#### 2.3.1. Tolerance to simulated gastric and intestinal fluid

Tolerance to simulated gastric and intestinal fluid was evaluated according to the method of Arboleya et al. [15]. Briefly, cells of isolated LAB from 16 h MRS culture were harvested by centrifugation (7,500 × g at 4 °C for 10 min), washed twice with phosphate buffered saline (PBS) pH 7.4 and resuspended in 500 µL of the same buffer. A 100 µL aliquot of cell suspension was added to 900 µL of simulated gastric juice (NaCl 125 mM, KCl 7 mM, NaHCO_3_ 45 mM and pepsin 3 g/L), with final pH adjusted to 2.5 or bile juice (45 mM NaCl, 1 g/L pancreatin and 3 g/L bile salt, adjusted to pH 8.0). Suspensions were then incubated at 37 °C for 2 h under anaerobic condition. The samples were aliquoted at time 0 and after 2 h. These samples were serially diluted in 0.85% NaCl and viable cells were counted by the spread plate method using MRS agar, and incubated at 37 °C for 48 h. The percentage of LAB survival was determined as follows:

$$\text{Survival}\left(\%\right)=\frac{\text{log CFU of viable cell after 2 h of incubation}\times \text{100}}{\text{log CFU of initial viable cell}}$$

Survival at more than 75% was considered tolerant to gastrointestinal conditions.

#### 2.3.2. Genotypic identification using 16S ribosomal DNA

Bacteria showing high tolerance to gastrointestinal conditions were molecularly identified using polymerase chain reaction (PCR), and pure cultures were sequenced by Macrogen Inc (Seoul, Republic of South Korea). Primers 785F 5′ (GGATTAGATACCCTGGTA) 3′ and 907R 5′ (CCGTCAATTCMTTTRAGTTT) 3′ were used to amplify the 16S rDNA gene. The PCR reaction was carried out with 20 ng of genomic DNA as the template in a 30 µL reaction mixture using an *EF-Tag* (SolGent, Korea) as follows: activation of Taq polymerase at 95 °C for 2 min, followed by 35 cycles of 95 °C for 1 min, 55 °C, and 72 °C for 1 min each, with a final 10-min step at 72 °C. The amplification products were purified with a multiscreen filter plate (Millipore Corp., Bedford, MA, USA). Sequencing reaction was performed using a PRISM BigDye Terminator v3.1 Cycle Sequencing Kit. DNA samples containing the extension products were added to Hi-Di formamide (Applied Biosystems, Foster City, CA, USA). The mixture was incubated at 95 °C for 5 min, followed by 5 min on ice, and then analyzed by an ABI Prism 3730XL DNA analyzer (Applied Biosystems, Foster City, CA, USA). A homology search was performed using BLAST software, and 16S rDNA sequences of the isolated LAB were deposited in GenBank.

#### 2.3.3. Autoaggregation and coaggregation assays

Autoaggregation and coaggregation assays were determined according to Re et al. [16]. Isolated LAB were cultured at 37 °C for 16 h in MRS broth. The cells were harvested by centrifugation (7,500 × g at 4 °C for 10 min), washed twice, resuspended in PBS buffer pH 7.4 and adjusted for optical density at 600 nm of 1.0. The suspensions were then incubated at 37 °C for 4 h and 0.1 mL of the upper suspension was aliquoted and added to 1.9 mL PBS. The suspensions were measured at OD600 nm, with percentage autoaggregation evaluated as follows:

$$\text{Autoaggregation}\left(\%\right)=\frac{\text{1}–\left({\text{OD}}_{\text{600}}\text{of upper suspension}\right)\times \text{100}}{{\text{OD}}_{\text{600}}\text{of total isolated LAB suspension}}$$

Coaggregation of isolated LAB and 8 pathogenic bacteria were investigated. Cell suspensions were prepared similarly to the autoaggregation evaluation. A 1 mL aliquot of isolated LAB was mixed with 1 mL of pathogenic strain and incubated at 37 °C for 4 h. The mixtures were measured at OD_600_ at time 0 and 4 h. Coaggregation percentage was calculated as follows:

$$\text{Coaggregation}\left(\%\right)=\frac{{\text{OD}}_{\text{600}}\text{of initial cell}–{\text{OD}}_{\text{600}}\text{of suspension after 4 h incubation}\times \text{100}}{\text{OD600 of initial cell}}$$

#### 2.3.4. Surface hydrophobicity

Surface hydrophobicity was measured according to Guan et al. [17]. The isolated LAB was grown in MRS broth at 37 °C for 24 h and the cell pellet was harvested by centrifugation. The cells were resuspended and washed twice in PBS buffer. The OD_600_ of suspension was adjusted to 0.5 ± 0.05, then 4 mL of cell suspension were mixed with 0.4 mL xylene or hexane, with PBS buffer used as the control. The aqueous phase was determined at OD_600_ after 1 h of incubation at room temperature. Surface hydrophobicity was calculated as follows:

$$\text{Surface hydrophobicity}\left(\%\right)=\frac{{\text{OD}}_{\text{600}}\text{of control}–{\text{OD}}_{\text{600}}\text{of suspension after 1 h incubation}\times \text{100}}{\text{OD600 of control}}$$

#### 2.3.5. Antimicrobial activity

Antibacterial activity of cell-free supernatant (CFS) of isolated LAB was investigated against eight pathogenic bacteria by the agar well diffusion method [18]. Overnight cultures of isolated LAB were collected by centrifugation (7,500 × g at 4 °C for 10 min). The CFS was divided into two groups. Group 1 was unadjusted for pH, while group 2 was adjusted to pH 6.5 using 1 N NaOH to eliminate inhibition from pH reduction. The CFS samples were filter-sterilized through 0.45 µm pore size filters.

Eight intestinal pathogens including *Escherichia coli* ATCC25922, *Staphylococcus aureus* ATCC 25923, *Salmonella* Typhimurium ATCC 14028, *Enterobacter aerogenes* ATCC 13048, *Enterococcus faecalis* ATCC 29212, *Enterococcus faecium* ATCC35667, *Shigella sonnei* ATCC 25931 and *Klebsiella pneumoniae* ATCC 13883 were grown in Mueller-Hinton broth at 37 °C for 24 h. The OD_600_ was adjusted to 1 and spread on nutrient agar. Then, a hole with a diameter of 5 mm is punched aseptically with a sterile cork borer and 30 µL of CFS were added to each hole and incubated at 37 °C for 24 h. The diameter of the inhibition zone was measured, with Streptomycin (10 µg/mL) used as the control.

#### 2.3.6. Antibiotic susceptibility

The antibiotic susceptibility of isolated LAB was assessed using the agar well diffusion method according to Tagg and McGiven [18]. The isolated LAB was spread on MRS agar and antibiotic solution containing penicillin (10 µg), ampicillin (10 µg), amoxicillin (30 µg), clindamycin (2 µg) and nalidixic acid (30 µg) was added to each well. The plates were incubated at 37 °C for 24 h and the inhibition zones were measured. Results were interpreted according to the cut-off levels proposed by Charteris et al. [19].

### 2.4. Statistical analyses

All experiments were carried out in triplicate. Statistical analyses were performed using SPSS Version 16.0 (SPSS Inc., USA). Duncan's multiple range test was used to determine the level of significant differences (*P* < 0.05).

## 3. Results and discussion

### 3.1. Isolation of LAB

Fifty colonies were isolated from 42 samples on MRS agar. Among these Gram-positive and catalase-negative strains, 26 strains were isolated from pla-ra obtained from all four markets, 19 strains were obtained from bamboo shoot pickles obtained from Chao Phrom and four Right Development markets, 3 strains from phak-gard-dorng obtained from Chao Phrom market and 2 strains from Thai fermented pork sausage obtained from four Right Development market. LAB were not observed from ginger pickle (Table 1). Results showed that pla-ra and bamboo shoot pickle were the main sources of LAB, concurring with Miyashita et al. [20] who isolated LAB from 144 kinds of pla-ra belonging to *Aerococcus viridans*, *Enterococcus avium*, *E. faecalis*, *E. faecium*, *E. hirae*, *E. thailandicus*, *L. plantarum*, *L. lactis*, *L. paracasei*, *Pediococcus pentosaceus*, *P. acidilactici*, *Tetragenococcus halophilus*, *W. cibaria*, *W. confusa*, *W. paramesenteroides* and *W. viridescens*. Rodpai et al. [21] also reported that *Tetragenococcus*, *Halanaerobium* and *Lactobacillus* were the dominant bacteria in pla-ra from Northeastern Thailand, while Thakur et al. [22], Lindayani et al. [23] and Mir et al. [24] isolated *Lactobacillus* spp. and *Streptococcus* spp. from bamboo shoot pickle and *L*. *plantarum* from fermented vegetables. All isolates were used to further investigate intolerance to simulated gastrointestinal conditions.

**Table 1.** Isolation of LAB from Thai tradition fermented food in Phra Nakhon Si Ayutthaya Province.

Market	Fermented food	Samples	Number	Isolates
Grand	Phak-gard-dorng	3	0	
	Bamboo shoots pickles	2	0	
	Pla-ra	3	5	H1, H2, H3, H4, H6

Chao Phrom	Phak-gard-dorng	4	3	S2, S4, S6
	Bamboo shoots pickles	4	13	K1, K2, K4, K5, K6, K8, K9, K10, L1, Q1, Q2, Q4, Q6
	Pla-ra	3	6	N4, N5, O5, P1, P3, P5

DD	Phak-gard-dorng	3	0	
	Bamboo shoots pickles	3	0	
	Ginger pickles	1	0	
	Pla-ra	5	10	a3, b6, f1, f3, f4, f6, f7, f8, f9, f10

4 Right Development	Phak-gard-dorng	3	0	
	Bamboo shoots pickles	3	6	k1, m1, m2, m3, m4, m5
	Ginger pickles	1	0	
	Pla-ra	3	5	q2, t1, t2, t4, t5
	Thai fermented pork sausage (Sai-krog-prieo)	1	2	x1, x2

Total		42	50	

### 3.2. Tolerance to simulated gastric juice and bile salt

High tolerance capabilities against simulated gastric juice and bile salt were associated with the bilayer membrane structure, which enables easy tolerance of inverse condition [25]. A 75% survival rate of LAB after 2 h of incubation in simulated gastric juice and bile salt was considered the cut-off level for tolerance. Fifty isolates from Thai traditional fermented food showed varied tolerance to simulated gastric juice and bile salt. Most isolates exhibited low survival in simulated gastric juice, and these isolates were not further tested. The remaining three isolates (a3, f4 and f8) from pla-ra and three isolates (K1, K4 and K9) from bamboo shoot pickle showed high tolerance to acidic condition with survival rate of 78.47–93.01%. Isolate f4 showed the strongest tolerance to simulated gastric juice in comparison with the other isolates (Table 2). Resistant to bile salt is a significant property of a potential probiotic because the small intestine and colon have high concentrations of bile salt that is toxic to cells. Most LAB isolates displayed tolerance to bile salt at higher than 75% survival, except isolates H4, H6, K5, K6, K8, S2, S4, t4, t5 and q2. Out of the 50 isolates, 6 were selected for identification with their higher tolerances to gastrointestinal tract conditions.

**Table 2.** Tolerance to simulated gastric juice and bile salt of LAB isolates

Isolate	Tolerance to gastric juice (%)	Tolerance to bile salt (%)	Isolate	Tolerance to gastric juice (%)	Tolerance to bile salt (%)
a3	78.47 ± 0.13	93.31 ± 0.32	m2	14.30 ± 0.14	97.87 ± 0.84
b6	13.84 ± 0.79	79.87 ± 0.58	m3	49.09 ± 0.08	99.87 ± 0.56
f1	70.90 ± 0.06	98.15 ± 1.17	m4	41.78 ± 2.18	86.65 ± 0.33
f3	64.33 ± 0.93	78.68 ± 0.13	m5	12.13 ± 0.46	99.44 ± 1.25
f4	93.01 ± 0.12	91.63 ± 1.84	N4	12.33 ± 1.56	99.84 ± 0.00
f6	54.51 ± 0.31	85.75 ± 0.02	N5	11.56 ± 0.78	95.36 ± 1.77
f7	73.61 ± 0.03	89.62 ± 0.89	O5	50.58 ± 0.44	93.51 ± 0.82
f8	83.42 ± 0.76	93.70 ± 0.35	P1	11.10 ± 0.16	91.09±1.68
f9	40.85 ± 1.64	94.46 ± 0.40	P3	55.89 ± 0.23	90.41 ± 1.24
f10	57.29 ± 0.16	99.19 ± 0.09	P5	10.92 ± 0.16	83.71 ± 2.18
H1	63.73 ± 1.02	89.98 ± 0.93	Q1	74.34 ± 0.32	93.78 ± 1.32
H2	46.78 ± 0.41	99.48 ± 0.33	Q2	70.42 ± 0.30	94.42 ± 2.12
H3	56.41 ± 1.59	91.91 ± 1.48	Q4	65.72 ± 0.66	99.13 ± 0.14
H4	63.75 ± 0.04	64.39 ± 1.72	Q6	69.69 ± 0.03	99.58 ± 0.27
H6	72.97 ± 0.42	55.43 ± 0.97	S2	9.21 ± 2.11	48.51 ± 2.49
K1	81.85 ± 0.25	95.21 ± 2.31	S4	11.48 ± 0.16	71.07 ± 0.87
K2	12.98 ± 0.35	95.93 ± 0.46	S6	10.25 ± 2.12	97.38 ± 0.18
K4	90.75 ± 0.05	94.62 ± 2.80	t1	44.32 ± 0.95	83.97 ± 0.42
K5	70.09 ± 0.19	55.11 ± 2.83	t2	11.52 ± 0.31	75.13 ± 1.31
K6	54.03 ± 0.56	67.32 ± 6.21	t4	43.96 ± 0.19	55.21 ± 3.22
K9	78.50 ± 0.06	89.94 ± 1.73	t5	42.18 ± 0.23	57.82 ± 2.12
K8	55.41 ± 0.33	19.63 ± 2.25	x1	49.70 ± 0.10	77.34 ± 1.22
K10	54.83 ± 0.07	98.88 ± 1.81	x2	46.88 ± 0.58	79.08 ± 0.56
L1	10.87 ± 0.16	96.39 ± 0.48	k1	10.21 ± 3.12	94.08 ± 0.67
m1	13.94 ± 0.18	83.39 ± 0.18	q2	9.23 ± 0.93	63.15 ± 3.95

### 3.3. Genotypic identification using 16S ribosomal DNA

Isolate a3 was identified as *Weissella thailandensis*, whereas isolates f4 and f8 were identified as belonging to *Enterococcus thailandicus* (Table 3). *W. thailandensis* and *E. thailandicus* were previously isolated from pla-chom and fermented sausage (mum) in Thailand, respectively [26],[27]. To the best of my knowledge, this is the first report of isolation of *W. thailandensis* and *E. thailandicus* from pla-ra. Isolates K1, K4 and K9 were determined as belonging to *Limosilactobacillus fermentum*, consistent with Chen et al. [28] and Behera and Balaji [29] who reported that *L. fermentum* was isolated from jiang-sun (fermented bamboo shoot) in Taiwan and dry fermented bamboo shoot in India, respectively. The high potential tolerance in simulated gastric juice of *E. thailandicus* isolates f4, f8 and *L. fermentum* isolates K1, K4 and K9 concurred with Hanghshenas et al. [30] who reported that *Enterococcus* and *Lactobacillus* strains showed high (51–82%) and moderate survival rates (71–76%) at low pH values, respectively. This finding suggested that these 6 isolates have the potential to survive in the human gastrointestinal tract and can likely survive passage through the stomach and small and large intestines.

**Table 3.** Identification of LAB with high tolerance in simulated gastric juice and bile salt.

Isolates	Genus	Identities (%)	GenBank ID of reference strain	Accession number
a3	*W. thailandensis*	99	LC097077.1	MZ577177
f4	*E. thailandicus*	99	LT223669.1	MZ577179
f8	*E. thailandicus*	100	LT223669.1	MZ577180
K1	*L. fermentum*	99	MK418588.1	MZ577182
K4	*L. fermentum*	100	MN589591.1	MZ577208
K9	*L. fermentum*	100	MN589591.1	MZ577210

### 3.4. Autoaggregation and coaggregation properties

The autoaggregation property of probiotics plays a major role in the colonization of host epithelial cells, resulting in improvement of host defense mechanisms against the gut [31]. Autoaggregation at higher than 40% is required for a potential probiotic strain [32]. Six LAB exhibited autoaggregation ranging from 93.40–95.01%, demonstrating good adhesion ability (Table 4). There were no significant differences in autoaggregation among the six isolates. Autoaggregation of *E. thailandicus* isolates f4 and f8 and *L. fermentum* isolates K1, K4 and K9 were 93.40–94.83% and 94.57–95.01%, respectively and higher than those of *Enterococcus* spp. from fermented sausage (29.0–67.0%) [33] and *Lactobacilli* from cocoa fermentation (31.18%) [34]. Results implied that these isolates can protect themselves from environmental stresses, and have the capacity to survive in gastrointestinal tract conditions [35]. Coaggregation is related to the ability to interact with other bacteria to form a barrier that prevents colonization by pathogenic bacteria. *W. thailandensis* isolate a3 showed higher coaggregation with other pathogenic bacterial LAB isolates, except with *E. faecalis* and *K. pneumoniae*, while *E. thailandicus* isolates f4 and *L. fermentum* isolate K4 and K9 had low coaggregation ability against pathogenic bacteria. All LAB isolates showed the highest coaggregation with *S. aureus* (10.15–38.50%), while *L. fermentum* K1, K4 and K9 aggregated with *E. coli* (5.56–8.62%) at a lower level than *L. plantarum* aggregation with *E. coli* O157:H7 (21.49–27.25%) [36]. Results indicated that isolate a3 prevented colonization of most pathogenic bacteria on the intestine.

**Table 4.** Autoaggregation and coaggregation ability of LAB isolates against pathogenic bacteria.

Isolate	Autoaggregation (%)	Coaggregation (%)							
		*E. coli*	*S. aureus*	*Salmonella* Typhimurium	*E. aerogenes*	*E. faecalis*	*E. faecium*	*S. sonnei*	*K. pneumoniae*
a3	95.00 ± 0.09*	11.73 ± 1.84^Ba^	38.50 ± 0.45^Aa^	14.36 ± 4.48^Ba^	4.95 ± 0.10^C*^	3.13 ± 0.39^Cc^	10.01 ± 2.92^Ba^	12.06 ± 1.14^Ba^	2.60 ± 0.41^Cd^
f4	94.83 ± 0.30*	5.13 ± 0.94^Cc^	12.94 ± 1.35^Acd^	12.13 ± 0.90^Aa^	5.86 ± 0.95^BC*^	8.08 ± 1.31^Ba^	5.46 ± 1.67^Cb^	4.97 ± 0.55^Cc^	5.73 ± 0.90^BCc^
f8	93.40 ± 0.33*	9.01 ± 0.32^Bb^	15.62 ± 2.66^Ac^	14.93 ± 3.35^Aa^	8.07 ± 1.84^B*^	5.72 ± 0.70^Babc^	9.11 ± 1.74^Bab^	2.24 ± 1.52^Cd^	8.25 ± 0.20^Bb^
K1	95.01 ± 0.28*	8.62 ± 0.81^ABb^	23.23 ± 3.76^Ab^	5.48 ± 1.56^Cb^	7.93 ± 1.62^BC*^	6.65 ± 0.27^BCab^	7.07 ± 1.56^BCab^	7.70 ± 0.73^BCb^	10.38 ± 0.69^Ba^
K4	94.57 ± 0.06*	7.15 ± 1.17^Bbc^	11.83 ± 0.38^Acd^	10.74 ± 1.95^Aa^	7.44 ± 1.30^B*^	4.35 ± 0.87^Cbc^	8.05 ± 1.16^Bab^	6.11 ± 0.10^BCbc^	6.24 ± 0.33^BCc^
K9	94.62 ± 0.15*	5.56 ± 0.74^Cc^	10.15 ± 2.07^ABd^	12.41 ± 2.17^Aa^	7.48 ± 0.17^BC*^	5.61 ± 1.81^Cabc^	5.76 ± 0.13^Cb^	5.69 ± 1.22^Cbc^	5.81 ± 0.07^Cc^

Lowercase letters indicate significant differences in percentage coaggregation among the 6 isolates against the same pathogenic bacteria (*P* < 0.05). Capital letters indicate significant differences in percentage coaggregation of each isolate against 8 pathogenic bacteria (*P* < 0.05).

*indicates no significant difference in percentage autoaggregation or coaggregation (*P* > 0.05).

### 3.5. Surface hydrophobicity

**Figure 1.**
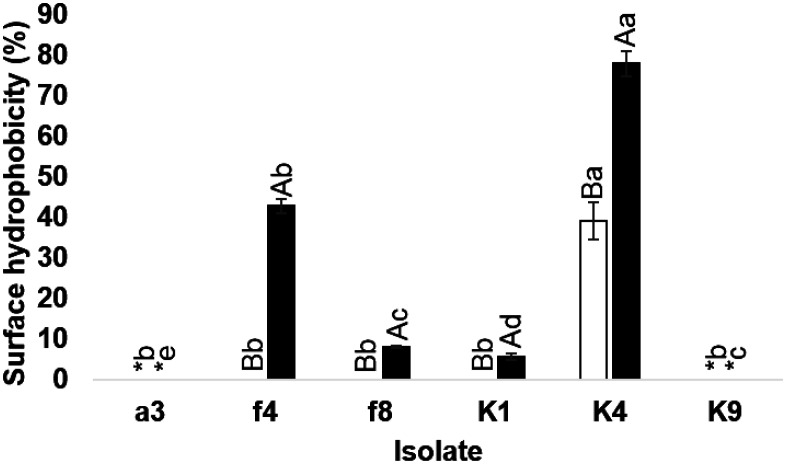
Surface hydrophobicity of LAB in the presence of xylene □ and █ n-hexane. Lowercase letters indicate significant differences in surface hydrophobicity percentages among 6 isolates in the presence of xylene or n-hexane (*P* < 0.05). Capital letters indicate significant differences in surface hydrophobicity percentages of each isolate in the presence of xylene and n-hexane (*P* < 0.05). *indicates no significant difference in surface hydrophobicity percentage (*P* > 0.05).

Significant differences in surface hydrophobicity values were found between LAB, even within the same species. *L. fermentum* isolate K4 showed high surface hydrophobic values as 39.15% and 78.09% with xylene and n-hexane, respectively (Figure 1). These values were lower and higher than Kocabay et al. [37], who reported surface hydrophobicity in the presence of xylene at 63.94% and n-hexane 57.59%, respectively. Surface hydrophobic values of *L. fermentum* isolate K4 also differed from Tamang and Tamang [38] who reported that *L. fermentum* isolated from fermented bamboo products of Arunachal Pradesh in India did not have a hydrophobic nature. Results indicated that *L. fermentum* isolate K4 may favor strong colonization through interaction with the organic mucin layer of the gut and play an important role in the adhesion of bacteria to epithelial cells [39]. *E. thailandicus* isolate f4 had surface hydrophobicity value of 42.83% in the presence of n-hexane, higher than reported by Santos et al. [40] who noted that *E. faecium* EM485 and *E. faecium* EM 925 had surface hydrophobicity values of 8.18% and 11.33% in the presence of n-hexadecane, respectively. Although, both xylene and n-hexane were non-polar solvents, most LAB isolates had higher surface hydrophobicity values with n-hexane than xylene, except *W. thailandensis* isolate a3 and *L. fermentum* isolate K9 that had surface hydrophobicity of 0% in the presence of both organic solvents. This finding differed from Xiong et al. [41] who reported that *W. confusa* possessed high surface hydrophobicity.

### 3.6. Antimicrobial activity

The antibacterial activity of CFS samples of all LAB disappeared when they were neutralized at pH 6.5 (data not shown). Results concurred with Zeng et al. [42] who reported that CFS from *L*. *plantarum* strains neutralized at pH 5.5 did not inhibit pathogenic bacteria. This finding suggested that antimicrobial activity of CFS derived from LAB was mainly due to organic acids such as lactic, formic and acetic acids that also play a role during growth in gastrointestinal tract conditions [43]. No CFS was obtained from 6 LAB that inhibited *S. aureus*, which is a Gram-positive bacteria because Gram-positive pathogens were less sensitive than Gram-negative pathogens (Table 5). This result concurred with Hartayanie et al. [2] who reported that *Lactobacillus* A1, A13, A17 and A19 isolated from bamboo shoot pickle had no antimicrobial capability against *S. aureus*, while Haghshenas et al. [30] noted that Gram-positive pathogens (*B. cereus* subsp. *Kenyae* PTCC 1539 and *E. faecalis* PTCC 1394) were more resistant than Gram-negative pathogens (*K. pneumoniae* PTCC 1053 and *Serratia marcescens* PTCC 1187). CFS samples without adjusting the pH obtained from *W. thailandensis* isolate a3 and *E. thailandicus* isolate f8 did not inhibit all pathogenic bacteria tested. *L. fermentum* isolates K9 and K4 showed the largest antimicrobial spectrum, exhibiting inhibitory activity against 4 and 6 pathogenic bacteria tests, respectively. This result concurred with Haghshenas et al. [30] who reported that *Lactobacillus* strains exhibited better anti-pathogenic activity due to excretion of organic acid, hydrogen peroxide, bacteriocins and biosurfactant.

**Table 5.** In vitro inhibition of pathogenic bacteria by LAB isolates.

Isolate	Diameter of inhibition zones (mm)							
	*Escherichia coli*	*Staphylococcus aureus*	*Salmonella* Typhimurium	*Enterobacter aerogenes*	*Enterococcus faecalis*	*Enterococcus faecium*	*Shigella sonnei*	*Klebsiella pneumoniae*
a3	0.00 ± 0.00^*d^	0.00 ± 0.00**	0.00 ± 0.00^*b^	0.00 ± 0.00^*c^	0.00 ± 0.00^*b^	0.00 ± 0.00^*b^	0.00 ± 0.00^*c^	0.00 ± 0.00^*c^
f4	0.00 ± 0.00^Bd^	0.00 ± 0.00^B*^	0.00 ± 0.00^B^	0.00 ± 0.00^Bc^	0.00 ± 0.00^Bb^	0.00 ± 0.00^Bb^	10.00 ± 0.00^Aa^	0.00 ± 0.00^Bc^
f8	0.00 ± 0.00^*d^	0.00 ± 0.00**	0.00 ± 0.00^*b^	0.00 ± 0.00^*c^	0.00 ± 0.00^*b^	0.00 ± 0.00^*b^	0.00 ± 0.00^*c^	0.00 ± 0.00^*c^
K1	7.50 ± 0.70^Ac^	0.00 ± 0.00^B*^	0.00 ± 0.00^Bb^	0.00 ± 0.00^Bc^	0.00 ± 0.00^Bb^	0.00 ± 0.00^Bb^	0.00 ± 0.00^Bc^	0.00 ± 0.00^Bc^
K4	9.00 ± 0.00^Cb^	0.00 ± 0.00^D*^	9.30 ± 0.50^Ba^	9.00 ± 0.00^Cb^	0.00 ± 0.00^Db^	9.00 ± 0.80^Ca^	9.00 ± 0.00^Cb^	11.00 ± 0.70^Aa^
K9	9.90 ± 0.80^Ba^	0.00 ± 0.00^C*^	0.00 ± 0.00^Cb^	10.00 ± 0.00^Aa^	10.00 ± 0.00^Aa^	0.00 ± 0.00^Cb^	0.00 ± 0.00^Cc^	10.00 ± 0.00^Ab^
Streptomycin	16.40 ± 1.50	17.00 ± 1.80	12.70 ± 0.50	21.00 ± 0.00	39.00 ± 1.10	23.20 ± 3.20	16.30 ± 3.70	18.70 ± 1.10

Lowercase letters indicate significant differences in the diameter of the CFS inhibition zone obtained from the 6 isolates against the same pathogenic bacteria (*P* < 0.05).

Capital letters indicate significant differences in diameter of the inhibition zone of CFS obtained from each isolate against the 8 pathogenic bacteria (*P* < 0.05).

*indicates no significant difference in the diameter of the inhibition zone (*P* > 0.05).

### 3.7. Antibiotic susceptibility

All LAB isolates were resistant against clindamycin and nalidixic acid, as antibiotics that inhibited protein synthesis and nucleic acid synthesis, respectively. They were also susceptible or moderately susceptible to ampicillin (Table 6). *W. thailandensis* isolate a3 and *E. thailandicus* isolates f4 and f8 exhibited resistance to penicillin. *W. thailandensis* isolate a3 had a different antibiotic susceptibility profile than the profile reported by Kamboj et al. [44]. They found that *Weissella* spp. exhibited susceptibility to penicillin and clindamycin, while *E. thailandicus* isolates f4 and f8 had different results from those reported by Wu et al. [45] who noted that *E. thailandicus* TC1 was found to be susceptible to penicillin. *E. thailandicus* isolate f8 showed high potential for resistance to 4 out of 5 antibiotics, whereas *L. fermentum* isolates K1, K4 and K9 only exhibited resistance to clindamycin and nalidixic acid. This result differed from EgervÄrn et al. [46] who indicated that *L. plantarum* exhibited low minimum inhibitory concentration (MICs) for ampicillin and clindamycin.

**Table 6.** Antibiotic resistant of isolated LAB.

Isolates	Antibiotic				
	Penicillin	Ampicillin	Amoxicillin	Clindamycin	Nalidixic acid
a3	R	S	MS	R	R
f4	R	S	MS	R	R
f8	R	MS	R	R	R
K1	MS	S	S	R	R
K4	MS	S	MS	R	R
K9	MS	S	MS	R	R

Susceptibility is expressed as R (resistant), MS (moderate susceptible) or S (susceptible).

## 4. Conclusions

Pla-ra and bamboo shoot pickle from markets in Phra Nakhon Si Ayutthaya were identified as major sources for isolation of LAB. Six isolating of LAB showing high tolerance to gastrointestinal tract conditions were identified as *W. thailandensis*, *E. thailandicus* and *L. fermentum*. *L. fermentum* isolate K4 showed outstanding probiotic properties such as tolerance to simulated gastric juice and bile salt with survival of 90.75 and 94.64%, high surface hydrophobicity, inhibition of various pathogenic bacteria and resistance to clindamycin and nalidixic acids. Isolate K4 showed potential as a candidate for further *in vivo* investigations to evaluate potential health benefits and safety assessment of probiotics for applications in the food industry.
